# Colles's Fracture

**Published:** 1892-05-28

**Authors:** 


### SURGERY.

### COLLES'S FRACTURE.

Ever since our remote ancestors ceased to walk on all-fours and stood erect, fractures of the lower end of the radius must have been common enough; and many learned men, no doubt, discussed the pathology of this lesion long before Colles investigated the subject. But it was not until the year 1814, when his article appeared in the *Edinburgh Medical and Surgical Journal*, that the peculiar deformity of the wrist we are so familiar with was definitely described and its causation indicated.

Since then there has been no dearth of literature on this injury, and it may still be said that there is room for more; for neither the manner in which the fracture occurs nor its exact nature can be said to be settled. For instance, immense labour has been expended on attempts to ascertain the seat of the fracture; and museum specimens have been appealed to and the results tabulated. Colles himself placed it one inch and a-half above the articular surface, but it undoubtedly varies, and is often much lower than this. In young people it is sometimes through the epiphyseal cartilage, for union with the shaft does not occur until the twentieth year. Whether it is transverse, oblique, or comminuted must depend on the direction of the force causing it, and on the condition of the bone, whether elastic, rigid, or fatty, &c. It may be noted that the outer layer of compact bone is particularly thin at the lower end of the radius, and the fractured surface of the lower fragment must be large and soft. We should, therefore, expect some crushing of this spongy, cancellous tissue, and impaction of the smaller (upper) into the larger surface. Bryant has examined the specimens of this injury in the museums of the London hospitals, and finds, in these, that impaction is usual. But we must remember that these dry specimens are almost always taken from bodies where the deformity of the wrist was so noticeable as to lead to a preservation of the bones, and must be looked upon as extreme examples, like most museum preparations. Many fractures of this kind are hardly recognisable as such after a few months, and these are not examined post mortem.

That impaction is common is obvious, for after the most successful treatment, when there is no possibility of the fragments over-riding each other, there is shortening. On the other hand, the locking of the bones is not firm enough, in most cases, to resist moderate attempts at reduction, provided the patient is under an anæsthetic. Without this it is not often that one can get a perfectly straight wrist, and patients, especially ladies, should always be warned that the best way of avoiding subsequent deformity in a severe Colles's fracture is to effect the reduction under chloroform or ether. This enables the surgeon to use sufficient force without fear of causing great pain, and it relaxes the antagonistic contraction of the numerous muscles round the bones.

As to the causation of the fracture. Direct violence is too rare a factor to be considered, but it is impossible to deny that such a thing is possible. Falls on the hands are certainly the commonest cause, and it has been noticed by more than one how frequent these accidents are upon the ice, when people usually fall with some force on to the out-stretched hands. Mr. Mansell Moullin points out that the fall is not usually with arms extended in front, but almost under the body. In skating, or on slippery roads the patient slips back and falls on the palm of the hand which is suddenly thrust downwards. This is important, because it shows, as Mr. Moullin observes, that a violent over-extension of the hand at the wrist joint occurs. The anterio radio-carpal ligament does not snap (as a rule), and there is consequently a "cross-breaking" strain put upon the bone. Again, Agnew remarks that if the weight of the body falls on the heel of the hand (*i.e.*, on the ball of the thumb, &c.) there is usually a comminuted fracture, owing to the fact that this implies a more vertical and therefore stronger strain on the radius. Whereas if the hand is so placed in falling that the force acts on the melacarpo-phalangeal points there is only a transverse "snap" of the bone. These statements have been verified by experiments in the post-mortem room, and show the importance of position, &c., in determining the nature of the injury.

It must not be forgotten that there is often a distinct displacement downwards of the ulna; its position with regard to the extensor carpiulnaris is altered, and the triangular fibro-cartilage may be torn from its surroundings. These lesions add considerably to the gravity of the injury and to the difficulty of reduction. Indeed, Professor Moore ("Transactions of the Medical Society of New York," 1870) considers this luxation of the ulna more important than the injury to the radius. He advises extension of the joint to be used in reduction combined with lateral and antero-posterior movements in order to disengage the extremity of the ulna from its new and abnormal surroundings. In most cases extension and abduction succeed, together with pressure over the dorsal and anterior prominences. When the limb is in a good position a splint should be applied with padding arranged where there was a prominence before reduction, but it is utterly useless to trust to the padding to effect the correction; it is only efficient to keep the bones in position, not to force them into it. Carr's splint is a good appliance, and the old-fashioned pistol-shaped splint gives good results, but both of them are somewhat deceptive, for the position of the hand implies more adduction to the ulnar side than really exists. Whatever splint is used it should not be kept on more than three or four weeks, and movements of the fingers should, of course, be begun long before this.

The weakness and aching which sometimes follows this injury, often try the patience of both surgeon and patient. Two practical points may be noted about this: firstly, that a very slight rheumatic tendency may be the cause of great pain in an injured bone or point; hence it is always worth while to try warmth, friction, and some internal remedies.

Secondly, it is important not to overdo the passive movements, and dull boring pains may often be relieved by a couple of days' perfect rest both of the arm and fingers, with a broad elastic wristband as a support.

